# Genome-wide identification and expression analysis of Ubiquitin-specific protease gene family in maize (*Zea mays* L.)

**DOI:** 10.1186/s12870-024-04953-5

**Published:** 2024-05-16

**Authors:** Weichao Fu, Delong Fan, Shenkui Liu, Yuanyuan Bu

**Affiliations:** 1 Key Laboratory of Saline-Alkali Vegetation Ecology Restoration (Northeast Forestry University), Ministry of Education, Harbin, 150040 China; 2 College of Life Sciences, Northeast Forestry University, Harbin, 150040 China; 3 State Key Laboratory of Subtropical Silviculture, Zhejiang A & F University, Lin’an, Hangzhou, 311300 China

**Keywords:** Maize (*Zea mays* L.), Ubiquitin-specific proteases (UBPs), Gene family, Phylogenetic analysis, Expression analysis

## Abstract

**Background:**

Ubiquitin-specific proteases (UBPs) are a large family of deubiquitinating enzymes (DUBs). They are widespread in plants and are critical for plant growth, development, and response to external stresses. However, there are few studies on the functional characteristics of the *UBP* gene family in the important staple crop, maize (*Zea mays* L.).

**Results:**

In this study, we performed a bioinformatic analysis of the entire maize genome and identified 45 *UBP* genes. Phylogenetic analysis indicated that 45 *ZmUBP* genes can be divided into 15 subfamilies. Analysis of evolutionary patterns and divergence levels indicated that *ZmUBP* genes were present before the isolation of dicotyledons, were highly conserved and subjected to purifying selection during evolution. Most *ZmUBP* genes exhibited different expression levels in different tissues and developmental stages. Based on transcriptome data and promoter element analysis, we selected eight *ZmUBP* genes whose promoters contained a large number of plant hormones and stress response elements and were up-regulated under different abiotic stresses for RT-qPCR analysis, results showed that these genes responded to abiotic stresses and phytohormones to varying degrees, indicating that they play important roles in plant growth and stress response.

**Conclusions:**

In this study, the structure, location and evolutionary relationship of maize *UBP* gene family members were analyzed for the first time, and the *ZmUBP* genes that may be involved in stress response and plant growth were identified by combining promoter element analysis, transcriptome data and RT-qPCR analysis. This study informs research on the involvement of maize deubiquitination in stress response.

**Supplementary Information:**

The online version contains supplementary material available at 10.1186/s12870-024-04953-5.

## Background

Ubiquitin-specific proteases (UBPs), the largest subfamily of plant deubiquitinating enzymes (DUBs), are involved in diverse physiological processes such as plant growth and development [1–5], as well as stress response [6–8]. According to previous studies, eukaryotic DUBs can be classified into five subfamilies and two types based on their catalytic domains: cysteine proteases and metalloproteinases [9, 10], with UBPs falling into the cysteine protease category [11]. UBP contains the Ub carboxy-terminal hydrolase (UCH) domain, which is mainly composed of two conserved motifs, cysteine cysteine (Cys) and histidine histidine (His) boxes, and plays an important role in the deubiquitination of plants [12]. Notably, the number of *UBP* genes varies significantly across species. For instance, the *Arabidopsis* (*Arabidopsis thaliana*) genome encodes 27 *UBP* genes [13], 25 genes in rice (*Oryza sativa*) [14], 48 genes in moso bamboo (*Phyllostachys edulis*) [15], and 97 in wheat (*Triticum aestivum*) [16]. The functions of *Arabidopsis UBP* genes have been extensively studied. Previous research has shown that *AtUBP3* and *AtUBP4* play crucial roles in male gametophyte development [1]. *AtUBP14* plays a role in the early embryonic development of plants [17]. *AtUBP26*, on the other hand, is involved in the ubiquitination modification process of histones and is essential for seed development [2, 18]. Furthermore, a recent study has revealed that *UBP15* plays a significant role in regulating seed development in both *Arabidopsis* and rice. *UBP15* modulates organ development and seed size in an opposing manner to the ubiquitin receptor DA1, and it positively regulates seed size by promoting cell proliferation in the maternal bead tissues [19]. OsUBP15 directly interacts with OsDA1 to positively regulate the length and width of rice seeds [20]. Loss of *AtUBP1* and *AtUBP2* function results in hypersensitivity of plants to the amino acid analogue canavaline (CAN) and severe dwarfing, short root development, and yellowing of leaves [13]. Overexpression of *UBP12*/*UBP13* can increase the NAC domain transcription factor *ORE1* level and positively regulate leaf senescence induced by nitrogen deficiency [4]. In addition, the deubiquitination enzymes UBP12 and UBP13 regulate the growth process of plants under nitrogen deficiency and positively regulate the recovery process after carbon starvation by regulating the stability of *Arabidopsis* BES1. In addition, UBP12 and UBP13 directly interact with RGF1 receptors to counteract RGF1-induced ubiquitination and promote root meristem development [21]. *UBP12* and *UBP13* also play an important role in the regulation of plant flowering time and the biological clock of plants [5]. UBPs have been shown to play an important role in stress responses, such as the plant immune response, drought response mediated by the ABA signaling pathway, salt response and other biological processes [6, 8, 22–24].

Maize is one of the world’s leading crops and is of considerable value to feed, food, pharmaceutical, and other industries [25, 26]. However, the *UBP* gene family in maize has not been extensively studied. To date, only three *ZmUBP* genes (*ZmUBP15*, *ZmUBP16* and *ZmUBP19*) have been characterized in maize [27]. *ZmUBP15*, *ZmUBP16* and *ZmUBP19* are the three homologous genes of *Arabidopsis UBP16* in maize, which play similar functions to *Arabidopsis UBP16* in response to salt stress. *ZmUBP15*, *ZmUBP16* and *ZmUBP19* expression levels are reduced under salt stress and partially rescue the salt-sensitive phenotype of *Arabidopsis ubp16-1* mutants, significantly enhancing the tolerance of *ubp16-1* mutants to salt stress [27]. In order to investigate the functions played by members of the maize *UBP* gene family in plant growth and development and stress response, we identified 45 *ZmUBP* genes in maize genome-wide, and their conserved motifs, gene structures, chromosome distributions, and expression patterns were analyzed. To understand their evolutionary relationship with other plants, a phylogenetic tree was constructed. Furthermore, the expression profiles of the *ZmUBP* genes under abiotic stresses and hormone conditions were assessed by using RT-qPCR. The findings of our study will help to understand the roles of *ZmUBP* genes in the stress response and to further identify the functions of this essential gene family in maize.

## Methods

### Identification of *UBP* genes in maize

The hidden Markov model (HMM) profile of the UCH domain (PF00443) obtained from the Pfam database (http://pfam.xfam.org/) was used to blast the maize protein sequence file using the local HMMER 3.0 program [28]. The E-value was limited to less than 1 × 10^−18^. All the identified *ZmUBP* candidates were verified using the Pfam database (http://pfam.xfam.org/). Proteins that did not have the UCH protein domain with highly conserved Cys residues (Cys-box) as well as His and Asp/Asn residues (His-box) were excluded. We then turned to the NCBI CD search (https://www.ncbi.nlm.nih.gov/Structure/cdd/wrpsb.cgi) to further annotate these genes. With the essential information on hand, we bioinformatically analyzed the *ZmUBP* genes using ExPASy (http://www.expasy.ch/tools/pi_tool.html). This analysis allowed us to determine the molecular weight (MW) and isoelectric point (pI) of the ZmUBP proteins. To gain a deeper understanding of their structural features, we predicted transmembrane structural domains using TMHMM (http://www.cbs.dtu.dk/services/TMHMM). Additionally, we utilized Plant-Ploc (http://www.csbio.sjtu.edu.cn/) to predict the hydrophobicity of the ZmUBP proteins.

### Analysis of *ZmUBP* gene structure and protein structure

To analyze the structure of *ZmUBP* genes, we used TBtools [29, 30] to compare CDS of the *ZmUBP* gene family with genomic DNA, mapped exon-intron structure, and predicted protein conserved motifs using MEME (https://meme-suite.org/). These motifs were mapped with TBtools [29, 30]. Finally, we combined the Gene Structure, protein conserved motifs, and protein domains based on the Gene Structure View (Advanced) function of Tbtools [29, 30].

### Sequence alignment and phylogenetic analysis

In this study, we retrieved 27 *Arabidopsis* UBP protein sequences from TAIR (https://www.arabidopsis.org/), 25 rice UBP protein sequences from Phytozome (https://phytozome.jgi.doe.gov/pz/portal.html), and 97 wheat UBP protein sequences from a previous study [16]. To investigate the evolutionary relationships among these proteins, we constructed a phylogenetic tree using the neighbor-joining method in MEGA 6.0 and visualized by iTOL (https://itol.embl.de/). To ensure the statistical reliability of our findings, we performed bootstrap testing with 1000 replicates.

### Analysis of cis-acting elements of *ZmUBP* gene promoters

To gain insights into the cis-elements in the promoter region of *ZmUBP* genes, we extracted the 2000 bp sequence upstream of these genes. We utilized the PlantCARE (http://www.dna.affrc.go.jp/PLACE/) to predict putative cis-regulatory elements in the promoter sequences. Notably, we identified functionally identical cis-acting elements that were uniquely named. To visualize the results of our analysis, we employed the Simple BioSequence Viewer function of TBtools [29, 30].

### Chromosome position, collinearity analysis and calculation of Ka/Ks ratios

We downloaded maize gff3 files from Ensembl Plants (https://plants.ensembl.org/) to analyze the annotation of *ZmUBP* genes on the chromosome. Using the maize genome annotation information, we obtained the relative distance and location of the *ZmUBP* genes on the chromosome. To visualize this data, we utilized the Gene Locatin Visualize feature of TBtools [29, 30].

The Dual Systeny Plot program in TBtools [29, 30] was utilized to assess the homology of *UBP* genes among maize and other species, including *Arabidopsis*, sorghum, soybean, Kinnow Mandarin (*Citrus reticulata Blanco*), cotton, medicago (*Medicago sativa*), rice, and wheat. To further analyze the collinearity and Ka/Ks ratio, we employed the One Step MCScanX and simple Ka/Ks calculator (NJ) of TBtools [29, 30], respectively. The results were visualized using the Microlife Letter (http://www.bioinformatics.com.cn/), providing a comprehensive understanding of the homology relationships among these species.

### KEGG and GO enrichment analysis

KEGG and GO enrichment analysis were performed using the microbiotics website (http://www.bioinformatics.com.cn/) to investigate the signalling pathways, biological processes, cellular components and molecular functions involved in the 45 *ZmUBP* genes.

### Temporal and spatial expression profiles of *ZmUBP* genes

We downloaded transcriptome data for different organs and stress responses of maize from the EMBL-EBI database (https://www.ebi.ac.uk/). To integrate the transcriptome data of the same type, we utilized EXCEL. The results were visualized and presented as heatmaps using the Microbiology Letter website (http://www.bioinformatics.com.cn/).

### Plant material, growth conditions, and stress treatments

Hybrid Zhengdan 958 was provided by Grain Crops Research Institute, Henan Academy of Agricultural Sciences (Validation No.: Guoshiyu 20000009, Date of Validation: 2000, Selection and Breeding Unit: Grain Crops Research Institute, Henan Academy of Agricultural Sciences, Selected Breeder: Chunxin Du, Variety Source: Zheng 58/Chang 7 − 2). To investigate the response of maize to various stress conditions, uniform-sized maize seeds were selected and sterilized with 75% ethanol for 5 min. The seeds were then rinsed five times with sterile water and placed in an incubator containing double layers of filter paper. The incubator was set to vernalize the seeds under a 12-hour light (25 °C) and 12-hour dark (20 °C) cycle. After 1 day of germination, well-established and uniform seedlings were selected and transferred into pots containing a mixture of vermiculite and soil (3:1 ratio). After 10 days of growth, the roots of the maize seedlings were watered with 100 mL of a 12% PEG 2000 solution to simulate drought stress. The seedlings were then exposed to low temperature (4 °C) and high temperature (42 °C) stress conditions. Additionally, hormone treatments were applied by watering the roots of the seedlings with different types of plant hormone solutions. All experiments were sampled on the whole plant at 0, 6, and 12 h, with at least three replications for each set of experiments.

### Reverse-transcription quantitative polymerase chain reaction

In order to study the expression characteristics of *ZmUBP* gene under different treatments, total RNA was extracted from the leaves of maize seedlings of control and treatment groups using OminiPlant RNA Kit from Beijing Kangwen Biotechnology Co. Subsequently, cDNA libraries were constructed using the Easy Script One-Step gDNA Removal and cDNA Synthesis Supermix from TransGen Biotech. Primers for the *ZmUBP* genes were designed using Primer 5.0 software. The cDNA from maize under different treatments was diluted 10-fold and used as a template, with the housekeeping gene actin serving as an internal reference. RT-qPCR was then performed to verify the gene expression characteristics under different treatments. The reaction system consisted of 1 µL cDNA, 1 µL Primer-FW, 1 µL Primer-RV, 10 µL 2× brilliant SYBR RT-qPCR master mix, and 7 µL ddH2O. The relative expression of the selected genes was calculated using the 2^-ΔΔCT^ method. RT-qPCR analysis was performed with three biological replicates.

### Statistical analysis

GraphPad Prism 9 was used to draw column and line charts, and SPSS 17.0 was used to analyze the significant differences (among the averages, there was no significant difference when there was a same marked letter, and there was significant difference when there were different marked letters, and the screening condition was *P* < 0.05). Pictures are mainly processed by Photoshop image processing software.

## Results

### Identification and characterization of the *UBP* genes in maize

To identify members of the *UBP* gene family in maize, we first searched relevant databases using the 27 *Arabidopsis* UBP protein sequences as queries, and this analysis identified 48 putative *ZmUBP* genes. The Pfam database (http://pfam.xfam.org) and NCBI CD Search (https://www.ncbi.nlm.nih.gov/Structure/cdd/wrpsb.cgi) were used to confirm the existence of the conserved domain UCH in these UBP proteins. After removing the unqualified sequences, a total of 45 *ZmUBP* genes were finally identified from the maize genome and named *ZmUBP1* to *ZmUBP45* according to their chromosomal locations (Table 1). All 45 ZmUBPs contained the UCH domain. Detailed information of *ZmUBP* genes were listed in Table 1, including gene length, physical and chemical parameters, and subcellular location of all *ZmUBP* genes. The length of *ZmUBP* genes conding sequence ranged from 1393 to 4462 bp, and the protein length of ZmUBPs was between 368-1284 amino acids. The molecular weight (MW) ranged from 41.81 to 187.82 kDa with the predicted isoelectric point (pI) ranging from 4.72 to 9.35. The transmembrane (TM) domains prediction of ZmUBP protein showed that only ZmUBP26 and ZmUBP22 contained one transmembrane domains. Subcellular localization and protein stability prediction showed that all 45 ZmUBP proteins were located in the nucleus and were unstable proteins. The hydrophobicity of 45 ZmUBP proteins was predicted to be less than 0, all ZmUBP proteins were hydrophilic proteins (Table 1).

**Table 1:** CDS length and protein sequence length, molecular weight, isoelectric point, protein hydrophobicity, transmembrane and domain analysis of 45 *ZmUBP* genes

Gene name	Gene ID	CDS (bp)	Protein length (aa)	Molecular weight (Da)	Transmembrane domains	Hydrophobicity	PI
*ZmUBP1*	*Zm00001eb006870*	1647	366	42.18	0	-0.459	5.97
*ZmUBP2*	*Zm00001eb008340*	3997	1073	119.35	0	-0.491	6.73
*ZmUBP3*	*Zm00001eb026810*	3293	890	100.29	0	-0.445	4.95
*ZmUBP4*	*Zm00001eb030930*	1811	456	53.09	0	-0.673	6.37
*ZmUBP5*	*Zm00001eb030950*	4030	1120	124	0	-0.712	7.06
*ZmUBP6*	*Zm00001eb038400*	3394	786	86.02	0	-0.644	6.85
*ZmUBP7*	*Zm00001eb068590*	2657	443	49	0	-0.449	8.17
*ZmUBP8*	*Zm00001eb073170*	1953	650	72	0	-0.556	8.41
*ZmUBP9*	*Zm00001eb081190*	1840	368	41.86	0	-0.413	6.37
*ZmUBP10*	*Zm00001eb082910*	1976	504	55.39	0	-0.028	6.97
*ZmUBP11*	*Zm00001eb090590*	3338	523	58.13	0	-0.267	5.57
*ZmUBP12*	*Zm00001eb102350*	3949	1061	115.9	0	-0.714	5.11
*ZmUBP13*	*Zm00001eb110870*	3362	552	60.33	0	-0.334	9.19
*ZmUBP14*	*Zm00001eb117210*	3408	1029	120.6	0	-0.614	5.52
*ZmUBP15*	*Zm00001eb132880*	4155	1114	130.72	0	-0.668	5.72
*ZmUBP16*	*Zm00001eb134530*	3495	443	50.39	0	-0.364	4.89
*ZmUBP17*	*Zm00001eb135380*	3939	1105	123.69	0	-0.781	6.29
*ZmUBP18*	*Zm00001eb151690*	4330	1232	137.05	0	-0.595	6.82
*ZmUBP19*	*Zm00001eb151920*	4080	1117	130.86	0	-0.651	5.97
*ZmUBP20*	*Zm00001eb162410*	1936	478	53.62	0	-0.505	5.97
*ZmUBP21*	*Zm00001eb175050*	3141	817	90.13	0	-0.624	5.11
*ZmUBP22*	*Zm00001eb180290*	3601	828	90.37	1	-0.623	7.34
*ZmUBP23*	*Zm00001eb184600*	5815	1693	187.82	0	-0.365	5.2
*ZmUBP24*	*Zm00001eb187750*	2555	560	62.39	0	-0.616	5.4
*ZmUBP25*	*Zm00001eb200320*	3619	1133	132.48	0	-0.574	5.81
*ZmUBP26*	*Zm00001eb204570*	3249	934	103.67	1	-0.515	6.92
*ZmUBP27*	*Zm00001eb225610*	2747	551	63.57	0	-0.661	6.44
*ZmUBP28*	*Zm00001eb238660*	3941	939	103.86	0	-0.48	7.88
*ZmUBP29*	*Zm00001eb244780*	1690	369	42.14	0	-0.402	7.29
*ZmUBP30*	*Zm00001eb251290*	2719	740	82.13	0	-0.592	5.1
*ZmUBP31*	*Zm00001eb274940*	3944	944	102.48	0	-0.424	5.52
*ZmUBP32*	*Zm00001eb292350*	4248	1319	145.89	0	-0.668	9.37
*ZmUBP33*	*Zm00001eb292450*	2713	644	70.82	0	-0.511	8.78
*ZmUBP34*	*Zm00001eb300350*	3242	874	97.11	0	-0.393	5.29
*ZmUBP35*	*Zm00001eb300680*	4261	1111	130.38	0	-0.641	5.86
*ZmUBP36*	*Zm00001eb310400*	4116	1056	117.48	0	-0.514	6.33
*ZmUBP37*	*Zm00001eb315400*	2798	707	78.39	0	-0.458	7.71
*ZmUBP38*	*Zm00001eb329890*	2475	552	60.18	0	-0.278	7.68
*ZmUBP39*	*Zm00001eb336890*	2985	795	88.53	0	-0.38	4.72
*ZmUBP40*	*Zm00001eb352840*	2861	658	72.76	0	-0.628	8.67
*ZmUBP41*	*Zm00001eb352890*	4462	1284	141.8	0	-0.723	8.86
*ZmUBP42*	*Zm00001eb374670*	3110	879	95.39	0	-0.539	9.35
*ZmUBP43*	*Zm00001eb390390*	3261	884	98.86	0	-0.707	5.85
*ZmUBP44*	*Zm00001eb408670*	4120	1121	131.35	0	-0.673	5.98
*ZmUBP45*	*Zm00001eb423870*	1393	368	41.81	0	-0.414	6.37

**Fig. 1:**
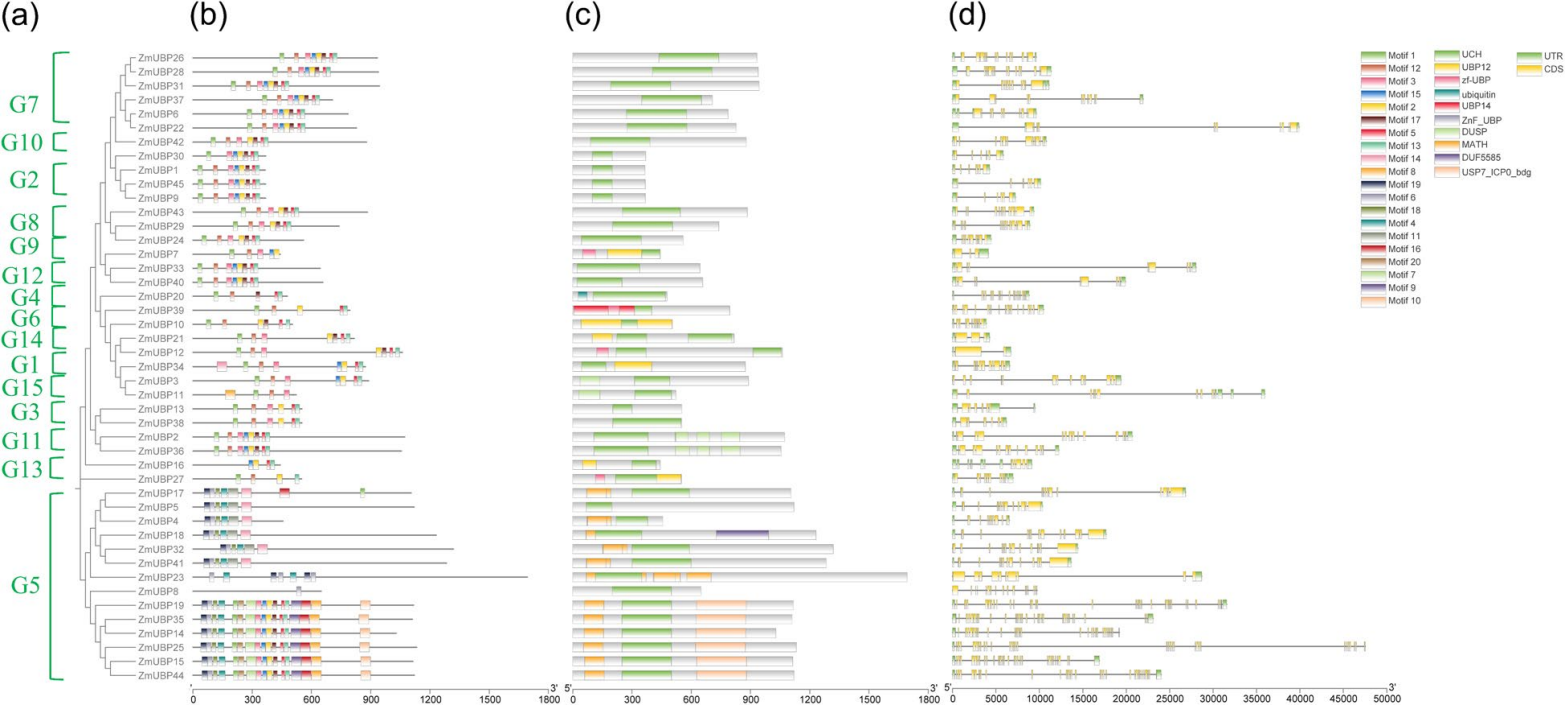
Gene and protein structures of 45 ZmUBPs. Phylogenetic relationships (**a**), protein motifs (**b**), protein structural domains (**c**) and gene structures (**d**) of 45 ZmUBPs. G1-G15 represent 45 ZmUBPs distributed in 15 different subfamilies, and the horizontal coordinates represent the length of genes/amino acid sequences

To better elucidate the association between gene function and evolution, we explored the structural organization and conserved motifs of *ZmUBP* genes (Fig. 1). The largest number of exons was 30, and they were detected in *ZmUBP25* from G5 subfamily, while the smallest number of exons was 2 detected in *ZmUBP12* from G1 subfamily. The exon number in other *ZmUBP* genes was between 3-26. The *ZmUBP* genes from the same subfamily shared comparable gene structure, thereby suggesting functional conservation among the maize *UBP* gene family. Additionally, the exon variation in *ZmUBP* genes may indicate the functional diversity of the maize *UBP* gene family. The MEME program was used to predict the composition of the ZmUBP protein motifs. A total of twenty conserved motifs were detected (Fig. 1). With few exceptions, the motifs in most ZmUBP proteins are arranged in the same order as motif 1, motif 2, motif 3, motif 15, motif 2, motif 17, motif 5, and motif 13 (Fig. 1).

### Phylogenetic analysis of the *UBP* genes in maize

**Fig. 2:**
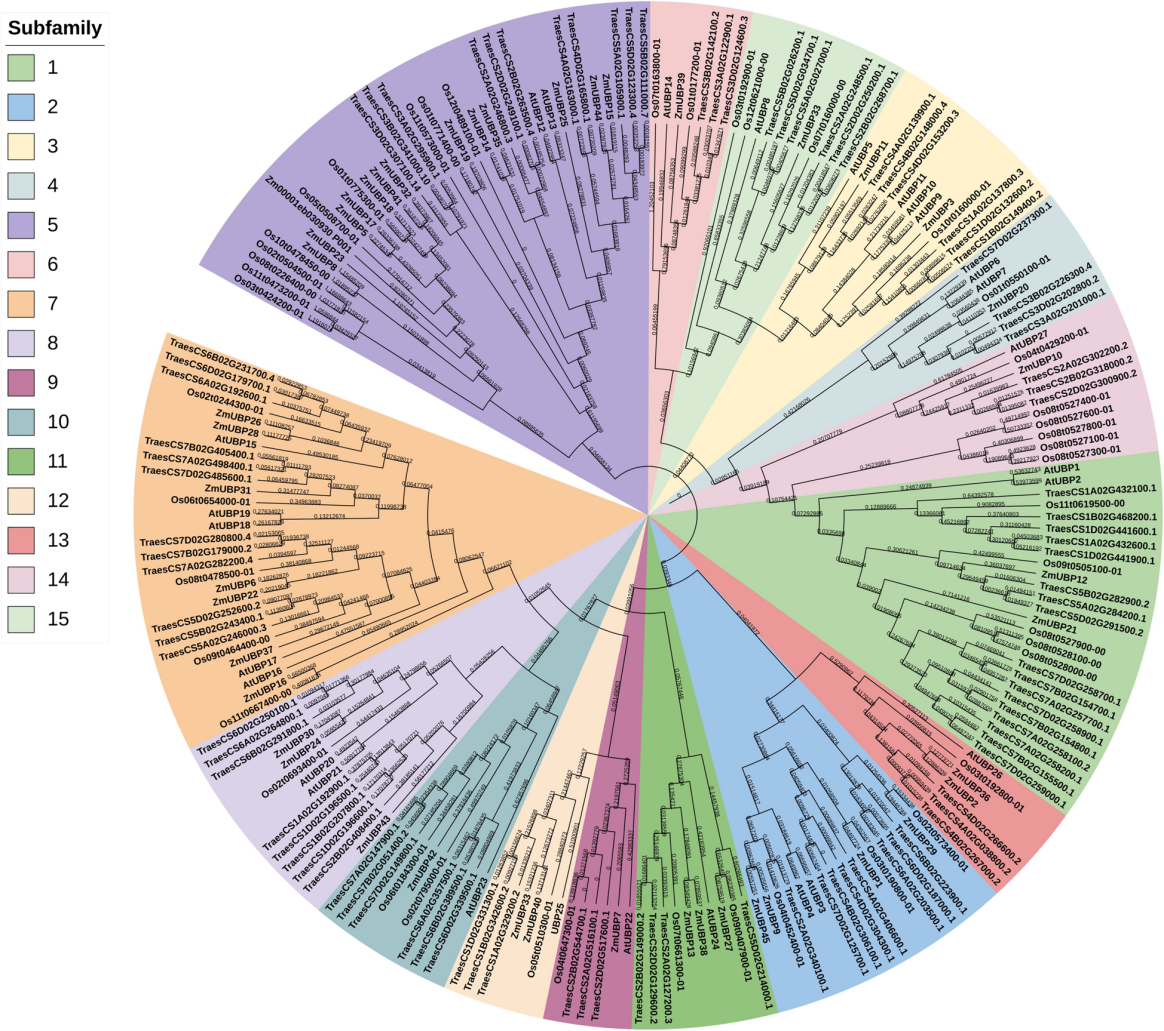
Phylogenetic relationships of UBPs from maize, *Arabidopsis*, wheat and rice. The phylogenetic tree was generated using a neighborhood join method with 1000 duplicates. Group 1-Group 15 on the left indicate that the *UBP* family members of maize, as well as the *UBP* family members of rice, wheat and *Arabidopsis*, are distributed in 15 different subfamilies. The branches of each subfamily are represented by a specific color. The branches of different members in the same subfamily have the same color

To analyse the phylogenetic organisation of the *UBP* family, we performed phylogenetic analysis using the protein sequences of 45 maize UBPs, 48 rice UBPs, 27 *Arabidopsis* UBPs, and 97 wheat UBPs, and generated a phylogenetic tree based on the Neighbour Joining (NJ) method (Fig. 2). Based on their phylogenetic relationships, we divided these UBPs into 15 groups, namely G1 to G15 (Fig. 2). G5 consisted of the most members, namely 14 ZmUBP proteins, followed by G7 including seven ZmUBP proteins. G4, G6, G9, G10, and G15 possessed the fewest members, including only one ZmUBP protein. Similarly, the UBPs of rice, wheat, and *Arabidopsis* were distributed among 15 groups, indicating that the functions of *UBP* family members were preserved during species evolution.

### Chromosomal location and evolution of *ZmUBP* genes

**Fig. 3:**
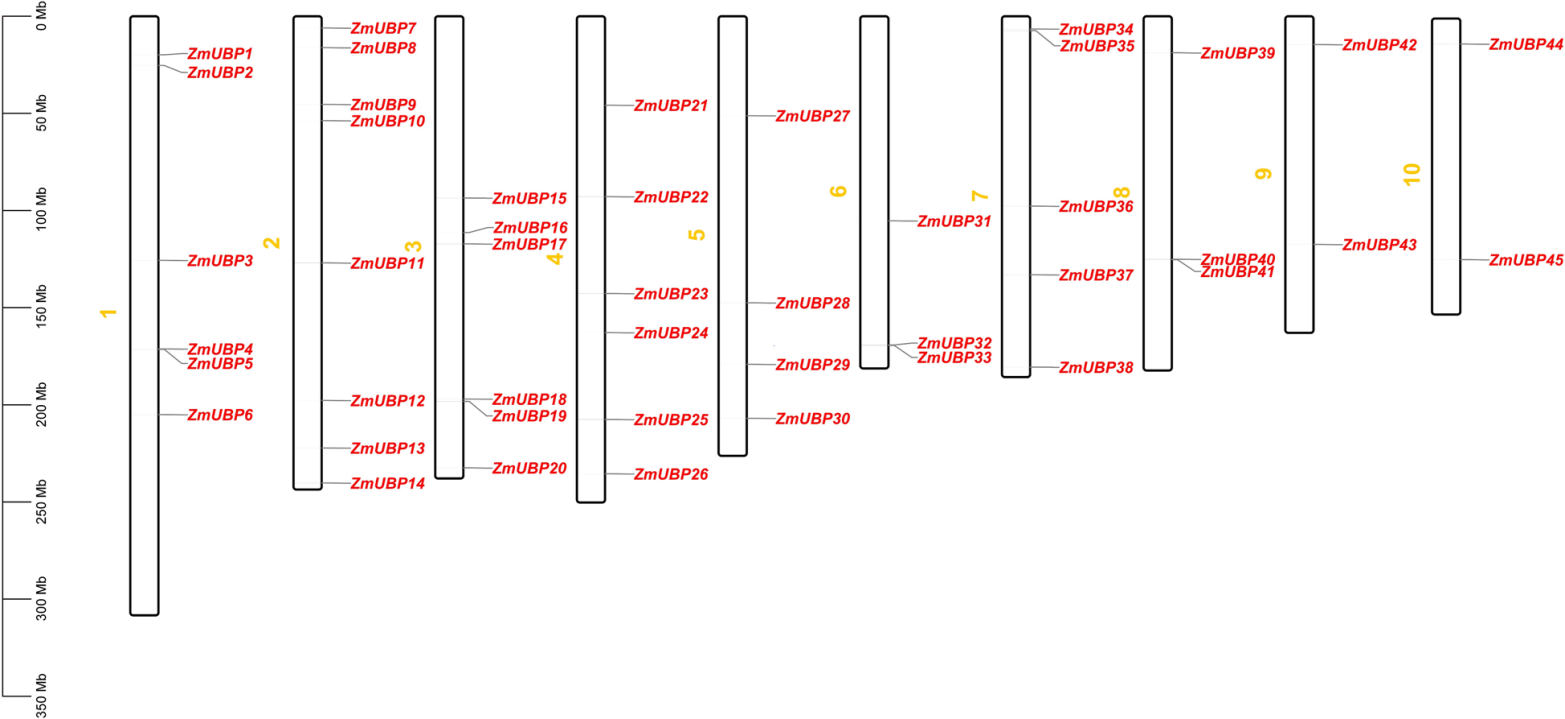
45 Distribution of *ZmUBP* genes on 10 chromosomes of maize. Each chromosome of maize has the distribution of *ZmUBP* genes, the vertical coordinate is the length of the chromosome, Zm1-Zm10 and the green columns indicate the 10 chromosomes of maize, and the red color represents 45 *ZmUBP* genes

To examine the chromosomal distribution of *ZmUBP* genes, the chromosomal location of each *ZmUBP* gene was determined. The results showed that the 45 *ZmUBP* genes were mapped to 10 chromosomes and they were unevenly distributed within each chromosome (Fig. 3). We identified 8 *ZmUBP* genes on chromosome 2. On chromosomes 1, 3, and 4, 6 *ZmUBP* genes were identified. On chromosomes 7, 5, 6, 8, 9 and 10, 5, 4, 3, 3, 2, and 2 *ZmUBP* genes were identified. These results indicate that there is genetic variation in the evolution of maize. Similarly, UBPs were unevenly distributed on different chromosomes in wheat [16], rice [14] and moso bamboo [15]. Obviously, the distribution of *UBP* genes on different chromosomes of different species is different.

**Fig. 4:**
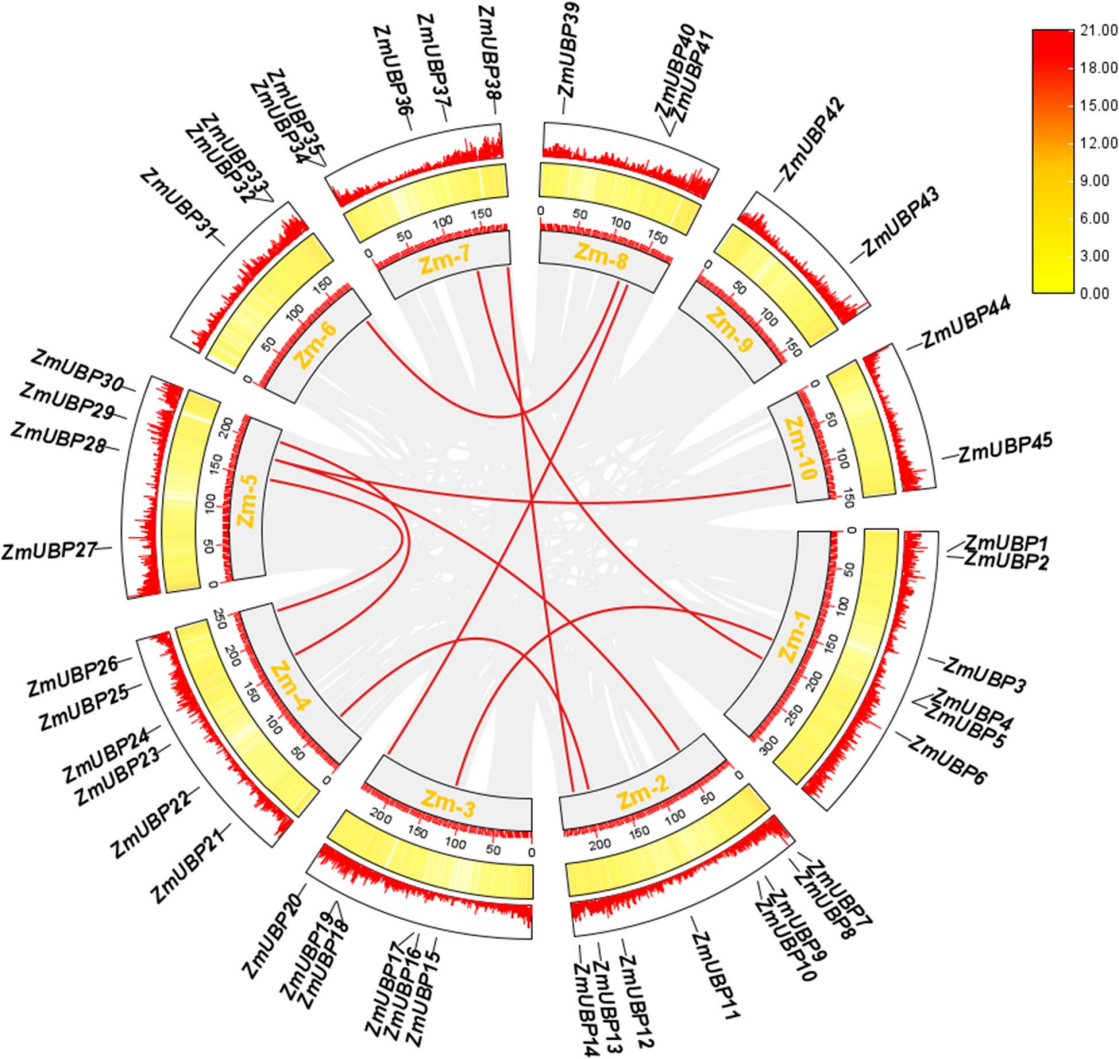
Duplication of 45 *ZmUBP* genes during evolution. Zm1-Zm10 in the innermost circle represents 10 chromosomes of maize, 0-150 represents the distance of genes on chromosomes, the yellow column represents the density of gene distribution on maize chromosomes, and the outermost circle is the distribution of 45 *ZmUBP* genes on chromosomes. The gray and red lines represent the gene duplication generated during the evolution of all genes in the maize genome and 45 *ZmUBP* genes, respectively

Duplication is the major impetus underlying gene expansion during evolution. Five types of gene duplication may occur in evolution, including singleton, dispersed, tandem, proximal, and segmental duplication [31]. Based on the chromosomal distribution analysis, we conducted gene duplication analysis to reveal the expansion process of the maize *UBP* genes (Fig. 4). The duplicate gene pairs were identified by comparing the coding sequences of 45 *ZmUBP* genes, and a total of 10 segmental duplication events (*ZmUBP5*/*ZmUBP16*, *ZmUBP6*/*ZmUBP37*, *ZmUBP9*/*ZmUBP29*, *ZmUBP12*/*ZmUBP21*, *ZmUBP20*/*ZmUBP41*, *ZmUBP24*/*ZmUBP30*, *ZmUBP26*/*ZmUBP28*, *ZmUBP29*/*ZmUBP45*, *ZmUBP33*/*ZmUBP40*, and *ZmUBP13*/*ZmUBP38*) were found in the maize genome (Fig. 4). These results indicate that segmental duplication plays an important role in *UBP* gene expansion in the maize genome. These results are consistent with the reported *UBP* members of monocotyledons such as wheat [16] and bamboo [15]. To investigate the evolutionary patterns among *ZmUBP* genes, we calculated the rate of synonymous substitutions between duplicate gene pairs. Our findings indicate that *ZmUBP* genes were generally under purifying selection, as evidenced by the Ka/Ks ratios of all duplicate gene pairs being less than 1 (Table 2).

**Table 2:** Nonsynonymous (Ka), synonymous (Ks)and Ka/Ks ratio between 10 duplicate gene pairs

Gene pairs		Ka	Ks	Ka/Ks
*ZmUBP6*	*ZmUBP37*	0.36	1.24	0.29
*ZmUBP4*	*ZmUBP16*	1.04	2.92	0.36
*ZmUBP9*	*ZmUBP29*	0.15	1.12	0.13
*ZmUBP12*	*ZmUBP21*	0.58	1.94	0.30
*ZmUBP13*	*ZmUBP38*	0.05	0.19	0.26
*ZmUBP24*	*ZmUBP30*	0.09	0.22	0.41
*ZmUBP26*	*ZmUBP28*	0.04	0.18	0.21
*ZmUBP29*	*ZmUBP45*	0.15	1.03	0.15
*ZmUBP32*	*ZmUBP41*	0.06	0.19	0.30
*ZmUBP33*	*ZmUBP40*	0.08	0.16	0.52

**Fig. 5:**
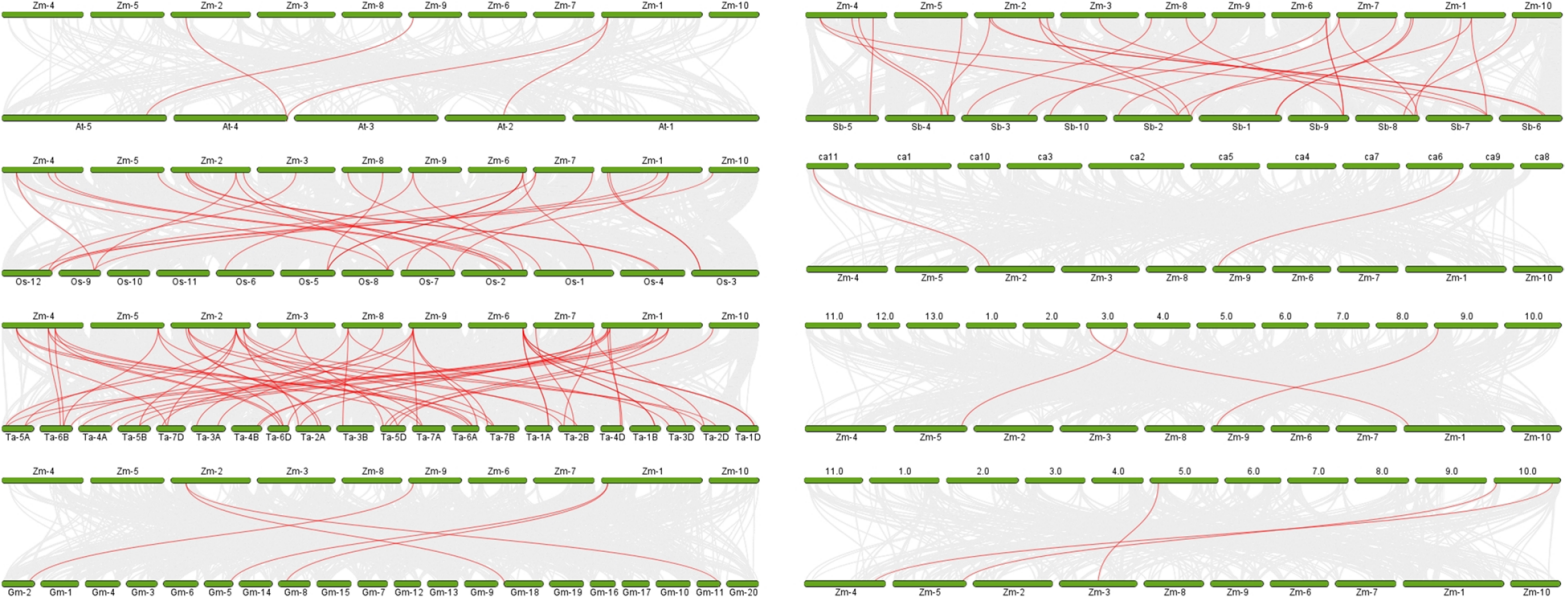
Collinearity analysis of *UBP *genes among maize, *Arabidopsis*, sorghum, soybean, Kinnow Mandarin, cotton, medicago, rice and wheat. The gray lines indicate the gene duplication that occurred during evolution between all genes in the maize genome and all genes in the genomes of eight different species. The red lines indicate gene duplication between the maize *UBP* genes and the *UBP* genes of eight different species that arose during evolution. The green columns represent the chromosomes of different species

To elucidate the evolution of the *UBP* gene families in maize, comparative syntenic maps were constructed with six dicotyledons, *Arabidopsis*, sorghum (*Sorghum bicolor*), soybean (*Glycine max*), Kinnow Mandarin, cotton (*Gossypium* spp.) and medicago, and two monocotyledons, rice and wheat (Fig. 5). The results of the comparison show that 20, 23, and 25 *ZmUBP* genes were covalently related to the *UBP* genes in sorghum, rice and wheat, respectively. This was followed by Kinnow mandarin, soybean, *Arabidopsis* and cotton, with three *ZmUBP* genes covalently related to their *UBP* genes, and only 2 *ZmUBP* genes were covalently related to the *UBP* genes of medicago. The *ZmUBP* genes had the largest number of covariate gene pairs with monocotyledons and much more than dicotyledons. These findings suggest that the *UBP* gene predates the evolution of dicotyledons and that genetic variation occurred during the transition from dicotyledons to monocotyledons.

**Fig. 6:**
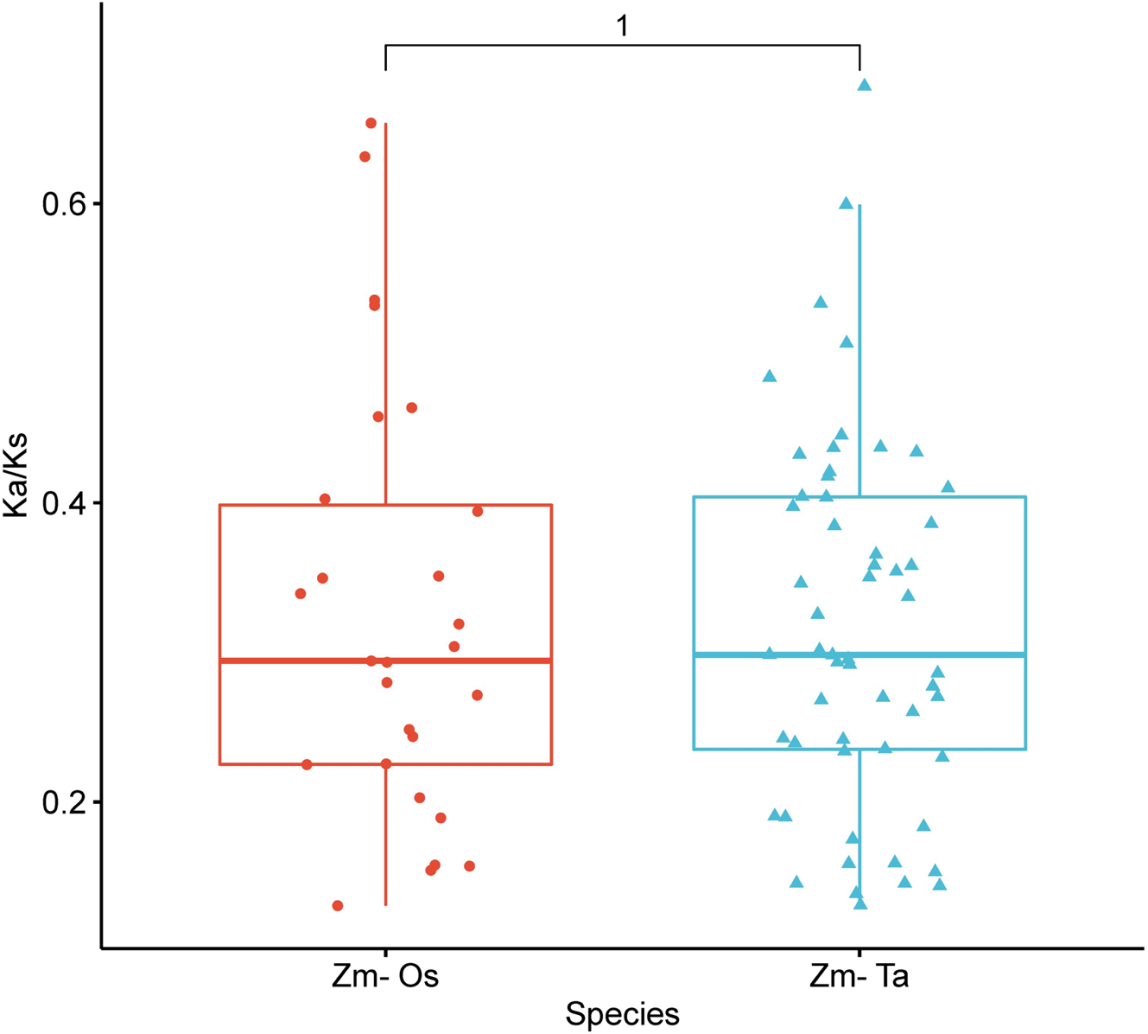
Ka/Ks ratios of homologous gene pairs in maize and rice (left) and wheat (right). The horizontal coordinates indicate the comparison of *UBP* genes between different species, and the vertical coordinates indicate Ka/Ks ratio ranging from 0-0.8. Red boxes and dots indicate homologous gene pairs between maize and rice, and blue boxes and triangles indicate homologous gene pairs between maize and wheat

To investigate the evolutionary patterns among *UBP* genes, we calculated the rate of synonymous substitutions between the *UBP* family in monocotyledon rice/wheat and maize (Fig. 6). The results showed that the Ka/Ks values of 27 homologous genes pairs between the maize *UBP* family and rice *UBP* family, as well as 56 homologous genes pairs between the wheat *UBP* family and maize *UBP* family were all less than 1 (Fig. 6), indicating that *UBP* genes were generally under purifying selection during the evolution of monocotyledons. This is consistent with the close evolutionary relationship between monocotyledons, suggesting that the *UBP* gene may play an important role in the evolutionary process of species.

### Analysis of *ZmUBP* genes related to stress response and plant hormone response

**Fig. 7:**
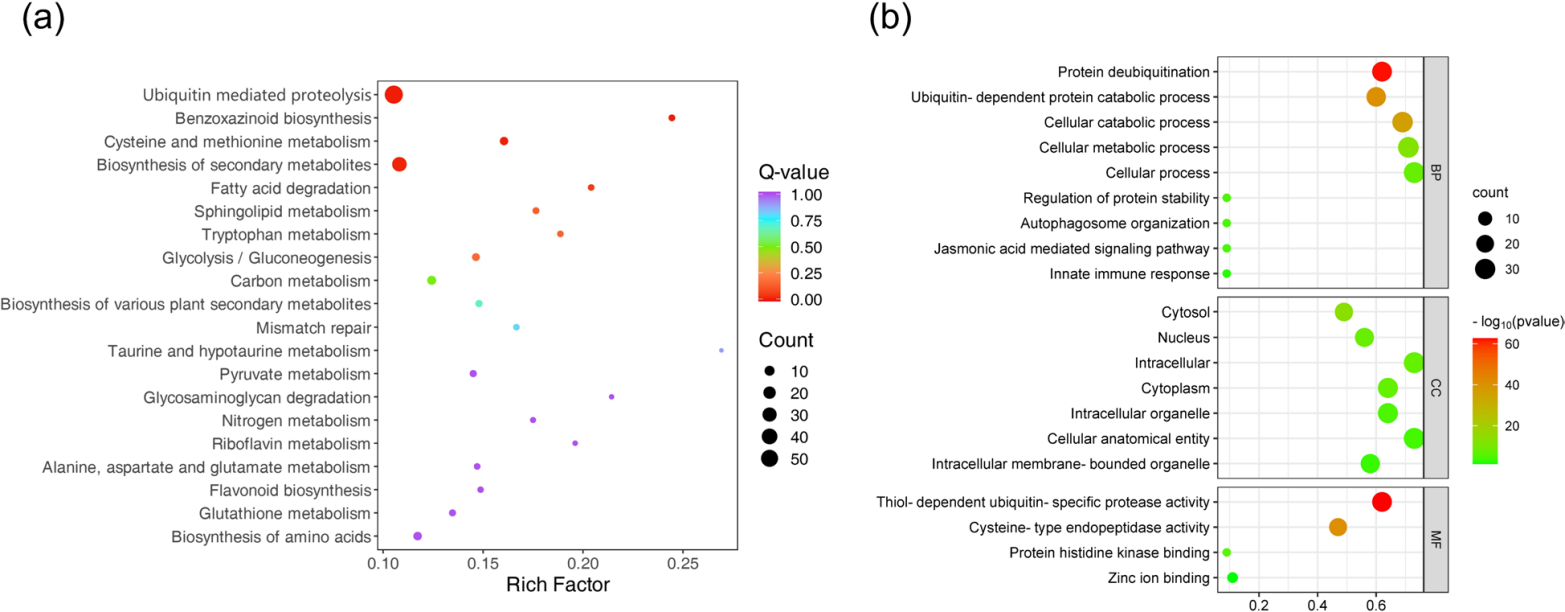
KEGG (**a**) and GO (**b**) enrichment analysis of the *ZmUBP* genes. The horizontal coordinates in **a** indicate the ratio of the number of genes in the pathway to the number of *ZmUBP* family members, and the vertical coordinates indicate the function of the *ZmUBP* family members. The colour of the circle indicates the significance of the proportion of genes in the pathway to the total genes (Q-value < 0.05 is significant). The size of the black circle indicates the number of *ZmUBP *genes in the pathway. The horizontal coordinates in ss **b** indicate the ratio of the number of genes in the pathway to the number of members of the *ZmUBP* family,and the vertical coordinates indicate the functions of *ZmUBP* family members, including cellular components, molecular functions and biological processes. The color of the circle indicates the significance of the proportion of genes in the pathway to the total genes (*P* < 0.05 is considered significant). After taking -log_10_(*P*value), the higher the value is, the higher the significance. The size of the black circle indicates the number of *ZmUBP *genes in the pathway

KEGG enrichment analysis showed that *ZmUBP* was mainly involved in ubiquitin-mediated protein hydrolysis and secondary metabolite synthesis processes (Fig. 7a). GO enrichment results showed that *ZmUBP* was mainly involved in biological processes such as protein deubiquitination, regulation of protein stability, autophagosome organization, jasmonic acid signaling pathway and immune response (Fig. 7b). This suggests that *ZmUBP* is involved in plant growth and stress response by regulating protein deubiquitination.

**Fig. 8:**
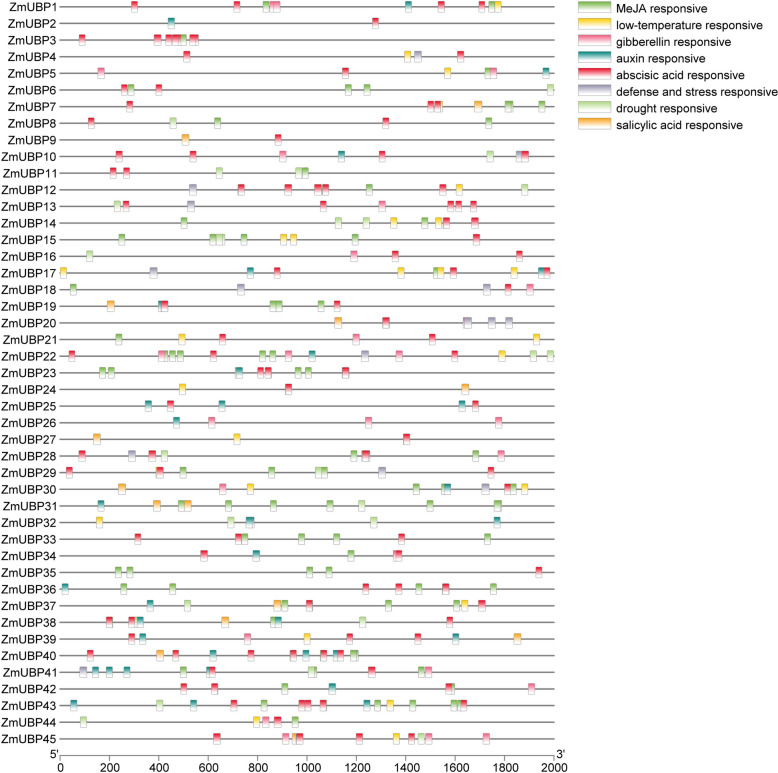
Cis-acting element analysis of 45 *ZmUBP* gene promoters. The 45 *ZmUBP* genes were classified according to phylogenetic relationships, and light, drought, gibberellin, jasmonic acid, abscisic acid, salicylic acid, auxin, stress, low-temperature, and seed growth response elements are indicated by 10 different colored columns. The horizontal coordinates represent gene length

The upstream regions of genes contain binding sites for promoters and transcription factors that regulate gene expression [32]. Therefore, the analysis of cis-acting elements contributes to the understanding of gene function and regulatory networks. To identify potential cy-acting elements in the promoter of *ZmUBP* genes, 2000 bp sequences upstream from the translation initiation codon of *ZmUBP* genes were retrieved and analyzed in the PlantCARE database. The promoters of the *ZmUBP* genes contain many elements or sites that respond to environmental factors such as light, temperature, and humidity. The presence of defense and stress response elements suggests that *ZmUBPs* may play an important role in the plant response to drought and low-temperature stress (Fig. 8). In addition, hormone response elements were found in the *ZmUBP* gene promoter, including abscisic acid (ABA), salicylic acid (SA), gibberellin (GA), auxin (IAA), and methyl jasmonate (MeJA) response elements. These results suggest that *ZmUBP* genes may be involved in the plant response to stress by regulating hormone signaling.

### Expression patterns of *ZmUBP* genes in different tissues

**Fig. 9:**
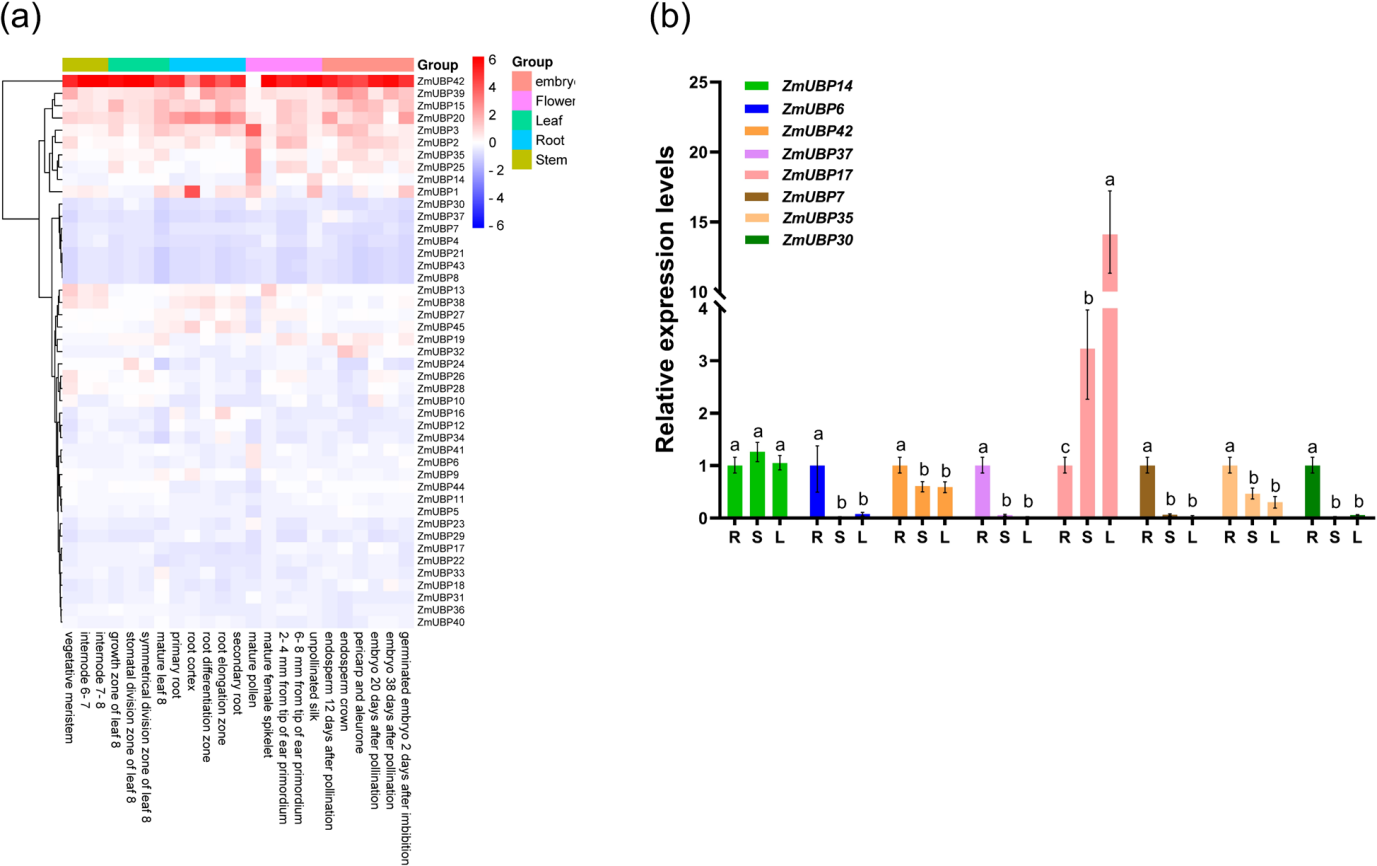
The temporal and spatial expression characteristics of *ZmUBP* genes. **a **Expression levels of 45 *ZmUBP* genes in 17 different tissues of maize. The horizontal coordinates represent different periods and different parts of the maize fetch, and the vertical coordinates indicate the clustering analysis of all *ZmUBP* genes according to the expression levels at different periods. The red and blue columns indicate the values after z-score normalization of the expression of *ZmUBP* genes, with red being expression above the mean and considered high expression, while blue being expression below the mean and considered low expression. Groups represent different parts of the plant, including root, stem, leaf, flower and embryo. **b** 8 *ZmUBP* genes that may play a role in plant growth and stress responses were selected based on the results of transcriptome analysis. Different colored columns represent different *ZmUBP* genes, and the horizontal coordinates represent different plant tissues, in which R stands for roots, S for stems, and L for leaves. The vertical coordinates represent the relative expression levels of the 8 *ZmUBP* genes in different plant tissues (using the expression in roots as control). Significant differences are indicated by the letters a, b, and c labeled at the top of the columns

The expression profiles of 45 genes in different tissues were divided into four groups (Fig. 9a). The first category includes *ZmUBP42*. Except for the low expression in mature pollen, the expression level of *ZmUBP42* in other maize parts was the highest among all *ZmUBPs*. This expression is similar to *AtUBP23* (AT5G57990), which is in the same subfamily as *ZmUBP42* in the evolutionary relationship (Fig. 2). *AtUBP23* is highly expressed in all tissues of *Arabidopsis* [33]. The second category includes *ZmUBP39*, *ZmUBP15*, *ZmUBP3*, *ZmUBP2*, *ZmUBP35*, *ZmUBP25*, *ZmUBP14*, *ZmUBP1* and *ZmUBP20*. Although the expression level of these genes is low in some plant tissues, the overall expression level is high (Fig. 9a). In particular, *ZmUBP39*, whose high-level expression during embryonic development is similar to *AtUBP14* (AT3G20630), which is in the same subfamily as *ZmUBP42* in the evolutionary relationship, further validates the conjecture in Fig. 2 that *ZmUBP39* may play an important role in early embryonic development. In addition, the expression level of *ZmUBP1* in reproductive organs was significantly higher than that in other parts, which was similar to *AtUBP3* (AT4G39910) and *AtUBP4* (AT2G22310) in *Arabidopsis*, which were in the same subfamily as *ZmUBP1* in the evolutionary relationship. This is the same as the speculation in Fig. 2 that *ZmUBP1* plays an important role in plant sexual reproduction. The third category includes *ZmUBP30*, *ZmUBP37*, *ZmUBP7*, *ZmUBP4*, *ZmUBP21*, *ZmUBP43* and *ZmUBP8*. These genes have low expression levels in all tissues of plants and may not be involved in plant growth and development (Fig. 9a). The expression levels of other genes in different plant tissues are quite different, *ZmUBP41* and *ZmUBP6* were highly expressed in mature pollen, *ZmUBP19* and *ZmUBP26* were highly expressed near the primordium, indicating that they may be involved in plant sexual reproduction. In addition, the expression levels of *ZmUBP* genes in maize roots and embryos were generally higher than those in other plant parts, indicating that *ZmUBP*-mediated deubiquitination may be crucial for nutrient uptake and sexual reproduction.

Up to now, the only study on maize *UBP* genes is the identification of three *Arabidopsis UBP* gene homologs in maize, namely, *ZmUBP15*, *ZmUBP16*, and *ZmUBP19*, which only analyzed their expression patterns in roots, leaves, spikes, and seeds, as well as in the presence of metal, salt, and osmotic stresses [25], but there is no in-depth study of the roles that all members of the maize *UBP* family play in plant growth and in response to high-temperature, low-temperature, and drought stresses. Therefore, we selected eight *ZmUBP* genes with high expression levels under abiotic stresses using RT-qPCR to investigate their roles in plant growth and abiotic stress response. The results showed that except for *ZmUBP17*, which was highly expressed in stems and leaves, the other 7 *ZmUBP* genes were highly expressed in roots (Fig. 9b), and the expression levels in roots were significantly higher than those in stems and leaves (except for *ZmUBP14*). This is consistent with the transcriptome data in Fig. 9a (the expression levels of *ZmUBP* genes was significantly higher in roots than in stems and leaves), suggesting that *ZmUBP* genes were mainly involved in plant root growth.

### Expression pattern of *ZmUBP* genes under abiotic stresses and phytohormone treatment

**Fig. 10:**
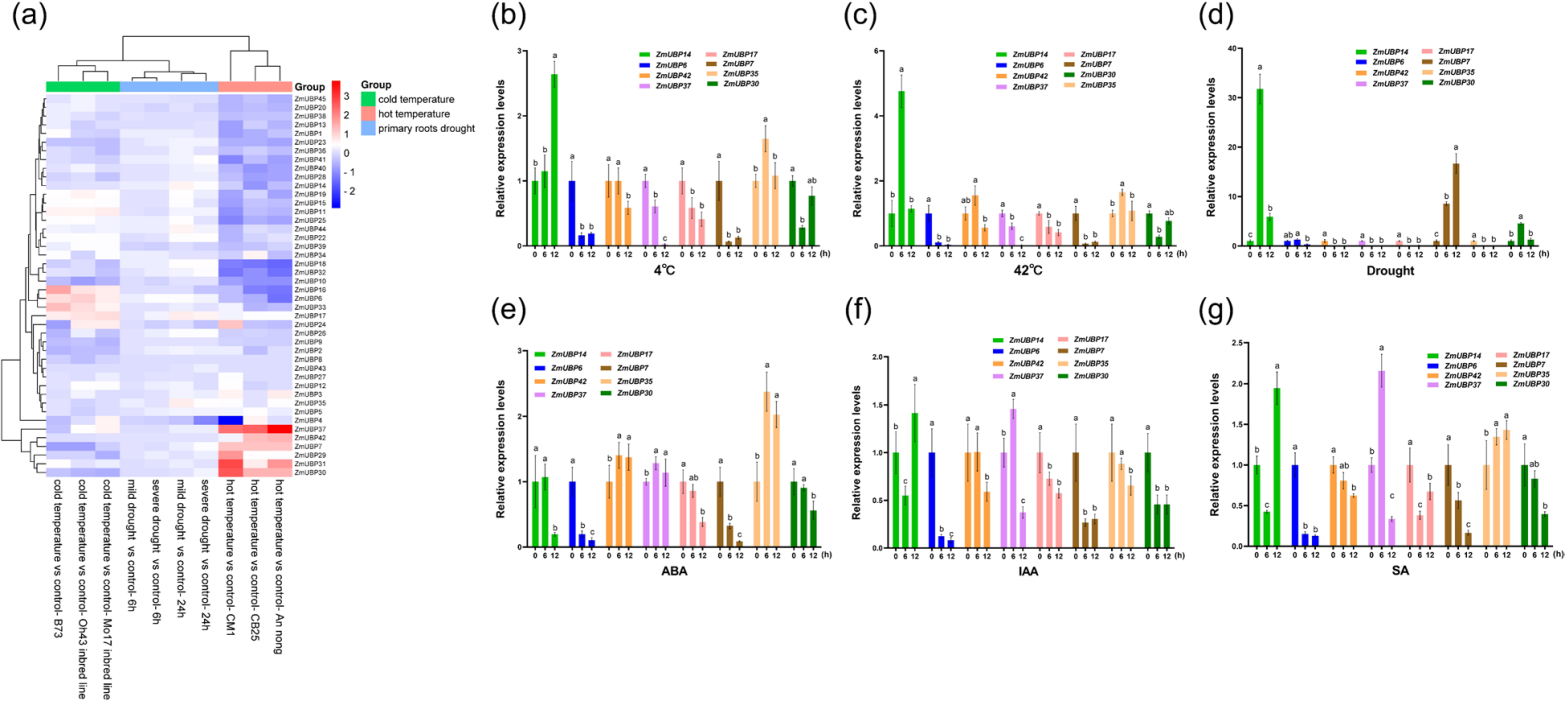
Expression analysis of *ZmUBP* genes in different tissues by RT-qPCR. **a** Expression levels of 45 *ZmUBP* genes under drought, low-temperature and high-temperature treatments. The horizontal coordinates represent the different treatments and treatment times of maize materials, and the vertical coordinates indicate the clustering analysis of all *ZmUBP* genes according to the expression levels of different treatments. The red and blue columns indicate the multiples of upregulated or downregulated expression of the treatment group compared with the untreated control group (red is upregulated expression, blue is downregulated expression). Group is different treatment methods of maize materials. **b-g** 8 *ZmUBP* genes that may play a role in plant growth and stress responses were selected based on the results of transcriptome analysis. Different colored columns represent different *ZmUBP* genes, and the horizontal coordinates represent abiotic stresses and phytohormones treatments for 0, 6 and 12 hours. The vertical coordinates represent the relative expression levels of the 8 *ZmUBP* genes under abiotic stresses and phytohormones treatments (using the expression under 0 hour as control). Significant differences are indicated by the letters a, b, and c labeled at the top of the columns

The expression levels of all *ZmUBP* genes were analyzed under abiotic stress (high-temperature, drought and low-temperature) based on transcriptomic data (Fig. 10a). The expression levels of all *ZmUBP* genes except *ZmUBP35* and *ZmUBP17* decreased significantly when maize taproots were subjected to drought stress (Fig. 10a), which is consistent with the results of promoter element analysis (Fig. 8), that is, most *ZmUBP* gene promoters contain drought response and ABA response elements, indicating that *ZmUBP* gene family members may be negative regulators of drought stress. The expression of *ZmUBP37*, *ZmUBP42*, *ZmUBP7*, *ZmUBP29*, *ZmUBP31* and *ZmUBP30* increased significantly after high-temperature stress was applied to different genotypes of maize (Fig. 10a). The expression of other *ZmUBP* genes was significantly downregulated. The expression multiples of *ZmUBP37* was most upregulated, which indicated that *ZmUBP37* was important for maize to cope with high-temperature stress. *ZmUBP16*, *ZmUBP6*, *ZmUBP33*, *ZmUBP11* and *ZmUBP17* were significantly induced in different varieties of maize after low-temperature stress. However, *ZmUBP24* was only upregulated in the resistant genotype, so *ZmUBP24* could be used as an important target for low-temperature resistance breeding of maize. In addition, the promoters of *ZmUBP22*, *ZmUBP1*, *ZmUBP7*, *ZmUBP10*, *ZmUBP34*, *ZmUBP27*, *ZmUBP32*, *ZmUBP35* and *ZmUBP15* also contained low-temperature response elements, but their expression levels were significantly reduced under low-temperature stress, indicating that these genes may be negative regulators of low-temperature stress. In addition, no *ZmUBP* gene was resistant to all three abiotic stresses.

RT-qPCR results showed that *ZmUBP14* was significantly induced after low-temperature, high-temperature and drought stresses, and the expression level showed a trend of decreasing and then increasing after IAA and SA treatments (Fig. 10b-d, f and g), suggesting that *ZmUBP14* may respond to abiotic stresses by cross-linking multiple phytohormone signaling pathways. In addition, the expression level of *ZmUBP35* was significantly increased after high-temperature, low-temperature and ABA treatments (Fig. 10b, c and e), suggesting that *ZmUBP35* may be an ABA-mediated temperature regulator. *ZmUBP37* was significantly induced by all three phytohormone treatments but did not respond to abiotic stresses (Fig. 10b-g), suggesting that *ZmUBP37* may regulate plant growth and development through a cross-linked phytohormone signaling pathway. Interestingly, *ZmUBP17*, which was highly expressed in roots, stems and leaves, did not respond to to either abiotic stress or phytohormones (Figs. 9 and 10), suggesting that *ZmUBP17* may affect plant growth and development through the regulation of other phytohormones. It is well known that ABA is a key phytohormone that regulates drought tolerance in plants, but no *ZmUBP* genes were found in this study that existed in response to both ABA and drought, suggesting that *ZmUBP* genes may regulate drought tolerance in plants through other pathways.

## Discussion

### Identification of *UBP* gene family members in maize

The eukaryote-specific *UBP* family is one of the largest *DUB* families identified to date and plays an important role in plant growth and development [34]. The plant *UBP* family has different types and amounts of *UBP* genes. There are 27 *UBP* members in *Arabidopsis* [13]. There are 48 *UBP* members in moso bamboo [15], and they are divided into two major groups (G13-G15, G1). There are 97 *UBP* members in wheat distributed in 15 subfamilies [16], and 48 *UBP* members in rice are also distributed in 15 subfamilies [14]. However, the *UBP* gene family members have not been identified in the maize genome. In this study, 45 putative *UBP* genes were identified in maize using genome-wide analysis, and were unevenly distributed on 10 chromosomes. According to phylogenetic analysis, the presumed *ZmUBP* gene family members were divided into 15 subfamilies as in wheat and rice, indicating that no *UBP* members were deleted during maize evolution. In addition, different members of the same subfamily are generally considered to have similar functions. *AtUBP3* (AT4G39910) and *AtUBP4* (AT2G22310) in *Arabidopsis* regulate male gamete development and affect plant sexual reproduction [1]. Therefore, *ZmUBP29*, *ZmUBP1*, *ZmUBP9* and *ZmUBP45*, which are in group 2, may also have the same function as *AtUBP3* and *AtUBP4*. Similarly, *ZmUBP39* in group 6 may be able to regulate plant early embryonic development like *AtUBP14* (AT3G20630). *AtUBP12* (AT5G06600) and *AtUBP13* (AT3G11910) have been shown to enhance plant drought tolerance through activation of the ABA signalling pathway and to play a role in plant histone debuquitination and organ development [35]. Therefore, *ZmUBP15*, *ZmUBP44*, *ZmUBP25*, *ZmUBP35*, *ZmUBP14*, *ZmUBP19*, *ZmUBP32*, *ZmUBP41*, *ZmUBP18*, *ZmUBP17*, *ZmUBP5*, *ZmUBP4*, *ZmUBP23* and *ZmUBP8* were also speculated to enhance plant drought resistance and to play a role in plant histone debuquitination and organ development because they also belong to the group 5. A recent study showing that The UBP5 histone H2A deubiquitinase counteracts PRCs-mediated repression to regulate *Arabidopsis* development informs the study of the histone deubiquitination function of the homologue ZmUBP5 in maize [36]. In addition, *OsUBP2* (Os09g0505100) in rice and *TaUBP1A.1* in wheat were proved to be resistant to leaf blight and Chinese wheat mosaic virus (CWMV), and both belonged to group 1 [6, 16], indicating that *ZmUBP12* and *ZmUBP21* belonging to group 1 subfamily may be resistant to biotic stress. In addition, *UBP* genes of wheat and rice were evolutionarily closer to maize *UBP* genes than *Arabidopsis UBP* genes (Fig. 2), as were interspecies covariance analyses (Fig. 5), confirming previously reported relationships between dicotyledons and monocotyledons during evolution [37]. These results indicated that *ZmUBP* genes existed before the isolation of monocotyledon plants and were highly conserved during plant evolution.

### Duplication events of *ZmUBP* genes during evolution

Gene duplication helps organisms adapt to environmental changes during development and growth and is essential for gene evolution and amplification [38, 39]. Among them, the tandem duplication of genes in the process of genomic DNA duplication and recombination is the key driver of gene family amplification [40]. In the genomes of *Arabidopsis* and rice, 15–20% of genes consist of tandem repeats of gene clusters thought to be critical for evolution, plant disease resistance, and abiotic stress responses [41]. Two tandem repeat clusters were found in the *TaUBP* gene family of wheat. The two tandem repeat genes were located on chromosomes 1D and 7D, accounting for only 3.7% of the 54 *TaUBP* collinear gene pairs, suggesting that tandem repetition may not be the main amplification method during the evolution of the *UBP* gene family.In the present study, chromosomal localization and gene structure revealed that gene duplication events occurred during genome expansion and evolution in maize. Forty-five members of the *ZmUBP* gene family produced 10 pairs of segmental duplicated genes without tandem duplication, with the same results as in wheat. It is further suggested that segmental duplication may be the primary method of gene amplification during the evolution of the *UBP* gene family, rather than tandem duplication. In addition, the Ka/Ks ratio can be used to determine whether selective pressure acts on protein-coding genes. In this study, the Ka/Ks ratio was significantly less than 1 in the intraspecific and interspecific repeat gene pairs of all maize *UBP* genes, indicating that strong purifying selection plays an important role in the constraint of *UBP* gene function, which is consistent with the conservation of the *UBP* gene in the evolutionary process.

### *ZmUBP* genes play an important role in plant growth and stress response

Transcriptional regulation of genes may be influenced by cis-elements in promoter regions that control responses to different stimuli. To investigate the biological function of *ZmUBPs*, we predicted cis-acting elements in the *ZmUBP* gene promoter. The results showed that the type of cis-acting element was different for each *ZmUBP* gene. Therefore, *ZmUBPs* may be involved in various specific regulatory mechanisms related to the stress response. Cis-acting regulatory elements largely determine tissue-specific gene expression patterns. In this study, we explored the expression profile of *ZmUBPs* in different tissues (root, stem, leaf, flower and embryo). The expression pattern of *ZmUBPs* showed that the expression levels of *ZmUBPs* were different in different plant tissues. The expression levels of *ZmUBPs* were the highest in maize roots and embryos and the lowest in leaves. This indicates that *ZmUBP*-mediated deubiquitination may be essential for the development of maize roots and embryos. In addition, some *TaUBPs* showed tissue-specific expression in maize, such as *ZmUBP42*, which was not expressed in mature pollen and was expressed at very high levels in all other sites, while *ZmUBP1* was only expressed at high levels in the root cortex and was expressed at low levels in other sites. These results indicate that *ZmUBPs* play different roles in plant growth and development. Cis-acting regulatory elements also largely determine the expression patterns of stress response genes. We found drought, low-temperature, GA, MeJA, ABA, and SA response elements in the promoter region of the *ZmUBP* genes, suggesting that *ZmUBPs* play an important role in the hormone-mediated stress response. This was confirmed by transcriptomic results. Except for *ZmUBP35* and *ZmUBP17*, the expression levels of all *ZmUBP* genes were significantly downregulated after drought treatment, indicating that *ZmUBPs* responded negatively to drought stress. The expression of *ZmUBPs* was different under low and high-temperature stress but showed a downward trend, indicating that *ZmUBPs* negatively regulated the plant stress response to abiotic stress.

Up to now, few studies have been conducted on the function of maize *UBP* genes. Kong et al. identified three *Arabidopsis UBP* gene homologs in maize and analyzed their expression patterns in roots, leaves, spikes, and seeds, as well as under metal, salt, and osmotic stresses [25]. However, the functions of *ZmUBP* genes in phytohormone, high-temperature, low-temperature, and drought responses have not been thoroughly investigated. In present study, we presented a comprehensive investigation of *ZmUBP* genes expression profiles in different plant tissues and abiotic stresses based on RT-qPCR and transcriptome data. Among them, *ZmUBP17* showed high expression levels in roots, stems and leaves, implying that it plays an important role in the growth and development of different parts of plants. Except for *ZmUBP17*, all other *ZmUBP* genes were expressed at high levels in roots and lower levels in stems and leaves. The process of deubiquitination has been shown to regulate nutrient uptake and transport in plant roots to maintain optimal root function, so we hypothesised that these seven *ZmUBP* genes also have the function of regulating nutrient uptake and transport in plant roots [42, 43]. In addition, we found that *ZmUBP14* was significantly induced by high-temperature, low-temperature, and drought treatments, and there was a positive response to IAA and SA, suggesting that *ZmUBP14* may regulate plant tolerance to abiotic stresses through an ABA-independent pathway. Interestingly, *ZmUBP17* showed a negative response to both abiotic stresess and phytohormones despite its high expression level in roots, stems and leaves, suggesting that *ZmUBP14* may regulate plant growth and development through other phytohormones besides ABA, IAA, and SA. ABA is a key phytohormone for regulating drought tolerance in plants, and ABA accumulation in the tissues can significantly enhance plant drought tolerance. However, this study did not find any *ZmUBP* genes that were responsive to both ABA and drought stress, suggesting that *ZmUBP* genes may regulate plant drought tolerance through an ABA-independent pathway. In addition, *ZmUBP37* was responsive to all three phytohormones, ABA, IAA, and SA, and was highly expressed in roots, suggesting that *ZmUBP37* may regulate the growth and development of plant roots by cross-linking multiple phytohormone signaling pathways. Combining the transcriptome and RT-qPCR results, we hypothesized that *ZmUBP14* may be a core member of the *ZmUBP* gene family that regulates plant growth and development and stress response.

## Conclusion

As the largest subfamily of plant *DUBs*, *UBP* is essential for deubiquitination and plays an important role in plant development and stress response. Here, we have conducted a genome-wide analysis of the *UBP* gene family in maize for the first time. A total of 45 *ZmUBP* genes were comprehensively identified from the maize genome, and all the maize *UBP* genes were randomly distributed on the 10 chromosomes of maize and produced 10 duplicate gene pairs in the evolutionary process. Phylogenetic analysis revealed that these maize *UBP* genes were divided into 15 subfamilies. The protein motifs and gene structures of the ZmUBPs were highly conserved in each group, reflecting their functional conservation. Collinearity analysis showed that a high proportion of the *ZmUBP* genes might be derived from tandem duplications with purifying selection, providing insights into possible functional divergence among members of the *ZmUBP* gene family. Furthermore, a large number of stress and hormone response elements are raised on the *ZmUBP* promoters. *ZmUBP14* was highly expressed in roots, stems, and leaves, and there was a positive response to drought, low-temperature, high-temperature, IAA, and SA, which has certain guiding significance for studying the mechanism of *ZmUBP* genes in response to abiotic stresses and hormones during development mediated by deubiquitination.

### Supplementary Information

**Supplementary Material 1.**

** Supplementary Material 2.**

** Supplementary Material 3.**

** Supplementary Material 4.**

** Supplementary Material 5.**

** Supplementary Material 6.**

** Supplementary Material 7.**

** Supplementary Material 8.**

** Supplementary Material 9.**

## Data Availability

Hybrid Zhengdan 958 provided by Grain Crops Research Institute, Henan Academy of Agricultural Sciences (Validation No.: Guoshiyu 20000009, Date of Validation: 2000, Selection and Breeding Unit: Grain Crops Research Institute, Henan Academy of Agricultural Sciences, Selected Breeder: Chunxin Du, Variety Source: Zheng 58/Chang 7-2). The genomic data analyzed in this study are available in the NCBI repository (the accession number of the maize genomic data is CABHLF000000000, the accession number of rice genomic data is JACJVL000000000, the accession number of sorghum genomic data is ABXC00000000, the accession number of soybean genomic data is ACUP00000000, the accession number of Kinnow Mandarin genomic data is NIHA00000000, the accession number of cotton genomic data is VKGJ00000000, the accession number of medicago genomic data is PSQE00000000, the accession number of wheat genomic data is NMPL00000000, The accession number of Arabidopsis genomic data is JAEFBJ000000000). All transcriptome data used in this study are available in the EMBL-EBI database (https://www.ebi.ac.uk/). Website links for all data used in this study are as follows. **Table:** 

Wibsite title	URL
Ensembl plants	https://plants.ensembl.org/
Pfam	Pfam: Home page (xfam.org)
NCBI CD search	NCBI Conserved Domain Search (nih.gov)
ExPASy	SIB Swiss Institute of Bioinformatics | Expasy
TMHMM	TMHMM 2.0 - DTU Health Tech - Bioinformatic Services
Plant-Ploc	Plant-PLoc server (sjtu.edu.cn)
SWISS MODEL	SWISS-MODEL (expasy.org)
MEME online tool	MEME - MEME Suite (meme-suite.org)
TAIR	TAIR - Browse - Gene Families (arabidopsis.org)
Phytozome	Phytozome (doe.gov)
iTOL	iTOL: Interactive Tree Of Life (embl.de)
PlantCARE	https://bioinformatics.psb.ugent.be/webtools/plantcare/html/
Microlife Letter	bioinformatics.com.cn
Tbtools	Releases CJ-Chen/TBtools (github.com)
STRING	STRING: functional protein association networks (string-db.org)
EMBL-EBI	https://www.ebi.ac.uk/

## Abbreviations

maize: *Zea mays* L.
UBPs: Ubiquitin-specific proteases
DUBs: Deubiquitinating enzymes
ABA: Abscisic acid
UCH: Ub carboxy-terminal hydrolase
Cys: Cysteine cysteine
His: Histidine histidine
*Arabidopsis*: *Arabidopsis thaliana*
rice: *Oryza sativa*
Moso Bamboo: *Phyllostachys edulis*
wheat: *Triticum aestivum*
CAN: Canavaline
CWMV: Chinese wheat mosaic virus
sorghum: *Sorghum bicolor*
soybean: *Glycine max*
Kinnow Mandarin: *Citrus reticulata Blanco*
cotton: *Gossypium *spp.
Medicago: *Medicago sativa*
Ka: Nonsynonymous substitution rates
Ks: Synonymous substitution rates
SA: Salicylic acid
GA: Gibberellin
IAA: Auxin
MeJA: Methyl jasmonate
